# Dr. Charles A. Meine

**Published:** 1915-08

**Authors:** 


**Dr. Charles A. Meine,** Buffalo 1870, died at his home in Germania, Pa., June 7, aged 85.

